# Analysis of willingness to use health management APP for female college students: application of UTAUT model based on Fogg theory

**DOI:** 10.3389/fpsyg.2024.1466566

**Published:** 2024-11-07

**Authors:** Lanying Wang, Yinying Zhang, Zhihong Li, Xinyu Pang, Yuanyuan Zhang, Mingming Zou

**Affiliations:** ^1^The Student Affairs Office of Dalian Medical University, Dalian, China; ^2^The Student Affairs Office of Liaoning Normal University, Dalian, China; ^3^Department of Chinese Medicine and Physiotherapy Dalian Rehabilitation, Recuperation Center of Joint Logistics, Support Force of PLA, Dalian, China; ^4^School of Public Health, Dalian Medical University, Dalian, China; ^5^Periodical Office, Dalian Medical University, Dalian, China

**Keywords:** health management apps, behavioral intention, female college students, UTAUT model, Fogg behavioral model

## Abstract

**Introduction:**

As the development process of medical industry informatization has entered the stage of smart healthcare, health management applications (apps) have played an important role in improving people’s health and preventing diseases, especially among female college students.

**Methods:**

This study combines the UTAUT model and the Fogg behavioral model (FBM) as a theoretical framework to investigate the factors affecting female college students’ willingness to use health management apps. A survey was conducted with 624 female college students regarding their usage of AI health management mobile applications.

**Results:**

The analysis reveals that social influence (*β* = 0.497, *p* < 0.001), performance expectancy (*β* = 0.268, *p* < 0.001), effort expectancy (*β* = 0.359, *p* < 0.001), and facilitating conditions (*β* = 0.603, *p* < 0.001) positively predict attitude; social influence (*β* = 0.36, *p* < 0.001) and effort expectancy (*β* = 0.183, *p* < 0.001) positively predict perceived risk, while facilitating conditions negatively predict perceived risk (*β* = −0.108, *p* < 0.01). Additionally, performance expectancy (*β* = 0.231, *p* < 0.001), effort expectancy (*β* = 0.285, *p* < 0.001), facilitating conditions (*β* = 0.25, *p* < 0.01), and attitude (*β* = 0.291, *p* < 0.05) positively predict an individual’s intention to use such applications, which in turn affects actual behavior (*β* = 0.804, *p* < 0.001).

**Discussion:**

This study develops a comprehensive theoretical framework to explore the psychological and social factors influencing female college students’ utilization of health management applications. The findings underscore the significant roles of social influence, performance expectancy, effort expectancy, and facilitating conditions in shaping user attitudes and intentions. These insights offer valuable guidance for formulating effective interventions to enhance the adoption of these applications.

## 1 Introduction

Health management is becoming increasingly important in modern society. With the acceleration of the pace of life and greater awareness of health issues, people are paying more attention to their own health status (Mano, 2021). The proliferation of smartphones and mobile applications has provided a convenient platform for personal health management, with health management applications (apps) becoming a popular tool (Kim et al., 2024; Wang and Qi, 2021). Health management app is a type of mHealth application, which is a health-related software program designed to run on mobile devices such as smartphones or tablets (Huang and Yang, 2020). It can provide users with a wide range of health management tools, including web-based consultation, health monitoring, diet management, exercise tracking, sleep analysis, health knowledge sharing, personal health management and other functions (Xu and Meng, 2024). The global digital health market is estimated to be $240.9 billion in 2023 and is expected to grow at a compound annual growth rate (CAGR) of 21.9% from 2024 to 2030 (Grand View Research, 2024). Additionally, the market for women’s health products and services is experiencing a period of rapid expansion. The global women’s health app market size was estimated at USD 4.14 billion in 2023 and is expected to grow at a CAGR of 17.7% from 2024 to 2030 (Grand View Research, 2024). These applications are utilized for the purpose of monitoring women’s health and bodily functions, including ovulation, pregnancy, breastfeeding, menstrual cycles, physical activity, mental health, emotional state, stress, and sleep (Alfawzan et al., 2022). With the proliferation of health management apps targeting the young female demographic, it is especially important to gain a comprehensive understanding of the available app options in the market, acquire practical insights, and identify the specific information and functionality that this user group seeks.

As a type of smart healthcare, health management app has emerged to provide patients with tools for self-monitoring, information access, and health management. The use of mHealth technology to improve health has been demonstrated in many diseases. Wang, L. G. found that with the help of portable mobile terminals, people can achieve efficient health management, so he researched mobile applications for personal exercise and health management with behavioral recognition and designed personal exercise and health management apps (Wang, 2017). Ramirez, V. et al. found that mHealth technology plays a significant role in improving the health outcomes of patients with chronic diseases, and it was concluded through investigation that even patients with lower socioeconomic status use cell phones with Internet and mobile app capabilities. Moreover, patients are willing to use mHealth technologies to manage chronic diseases and improve their overall health status (Ramirez et al., 2016). Bretschneider, Maxi Pia, et al. demonstrated that Vitadio may be effective in supporting patients’ diabetes management by motivating them to adopt healthier lifestyles and improve self-management by examining the impact of the health app Vitadio on improving glycemic control in type II diabetes (Bretschneider et al., 2022). Jensen, Victor Vadmand, et al. found that the use of mHealth in Denmark is growing, and their respondents generally believe that mHealth use is beneficial and related to their frequency of use (Jensen et al., 2023).

It is well documented that physical and mental health problems are prevalent among college students, with female students being especially vulnerable (Shin et al., 2023). Lifestyle modifications, including dietary habits, stress management, and alcohol consumption, have been demonstrated to influence hormonal balance in women (Grand View Research, 2024). Numerous studies have demonstrated that female college students tend to exhibit elevated levels of anxiety relative to their male counterparts. Additionally, there are notable gender differences in the prevalence and severity of mental disorders and physical activity-related issues (Glavin et al., 2021; Ma et al., 2022). The advent of the digital age has witnessed the proliferation of health management apps, which offer college students convenient access to health-related information and resources that can influence their overall health status and health-related behavior choices (Basch et al., 2018). A review of the literature reveals that female users are more likely than males to utilize apps pertaining to nutrition, self-care, activity tracking and reproductive health (Bol et al., 2018; Haluza and Wernhart, 2019). Despite the significant potential of health management apps for promoting health and preventing disease, and the fact that highly educated individuals and women are more likely to use mHealth apps, there is a relative lack of research on the factors that influence the sustained use of these apps by college women. Therefore, an in-depth study of the factors shaping female college students’ willingness to use health management apps and their behaviors will not only support the promotion of healthier individual behaviors but also help app developers refine the design and marketing strategies of their apps to better meet the needs of their target audience. This, in turn, can further enhance the role of these apps in health promotion and disease prevention, ultimately contributing to the improved overall health of young women.

The use of persuasive techniques in mobile applications has the potential to influence user behavior (Idrees et al., 2024; Matthews et al., 2016). The Fogg Behavioral Model (FBM) is a theoretical framework developed by B.J. Fogg for the purpose of assessing the process of persuasive techniques. It is comprised of three elements: motivation, ability and triggers. The core idea is that a person adopts a certain behavior depending on their motivation, their ability to perform the behavior, and what triggers the behavior (Fogg, 2009). It not only simplifies and illuminates the mechanisms of behavioral change, but also supports the understanding of the complexity of behavior as a framework for analyzing and designing behavior. In addition, research on the application of the FBM model among female college students is relatively scarce. This study employs the FBM model to uncover the behavioral motivations behind female college students’ use of health management apps, providing a theoretical foundation for understanding certain complex behaviors. Thus, using the FBM to predict the behavior of female college students in using health management apps is innovative and offers a new perspective for behavioral research on this demographic. However, although the FBM provides a clear structure to the process of behavioral change, this simplification may overlook other important factors such as the broader influence of society and the environment (Wu and Gong, 2023). The Integrated Technology Acceptance and Use Model (UTAUT), initially proposed by Venkatesh et al. (2003), integrates multiple factors into a unified theoretical framework with the objective of explaining and predicting users’ willingness to adopt new technologies and their actual use behavior, and examines the influence of multiple factors on these behaviors (Venkatesh et al., 2003). The UTAUT model has been subjected to empirical testing in numerous studies (Abbad, 2021; Abdekhoda et al., 2022; Hsu and Peng, 2022; Wu et al., 2022). The model integrates variables such as social influence and facilitating conditions to highlight the impact of psychosocial factors and the technological environment on technology acceptance and use, while also considering how both internal and external factors shape users’ behavior in adopting technology (Chao, 2019; Fretzen, 2018; Qiu et al., 2023). The UTAUT model and FBM explain user behavior and its driving factors from different perspectives. Combining the two can provide a more comprehensive analysis of female college students’ behavior when using health management apps. However, few studies have integrated FBM with UTAUT to analyze user behavior. Therefore, analyzing user behavior in health management apps from the perspective of combining FBM and UTAUT not only contributes to theoretical expansion and deepening but also provides practical guidance for application, promoting the further development and implementation of health management technologies on mobile platforms.

Therefore, this study focuses on female college students as the research subjects. By integrating the Fogg Behavioral Model and the UTAUT Model, an effective theoretical framework is established to investigate the factors that influence female college students’ intention and behavior regarding the utilization of health management applications. Based on the results of this study, we propose recommendations for the design of future health management apps. These recommendations are intended to assist developers in improving their products to better align with the diverse health management needs of female college students. Additionally, they seek to cultivate students’ awareness of health management, enhance their ability to utilize medical services, and ultimately improve their quality of life.

## 2 Method

### 2.1 Theoretical framework

In this study, a research hypothesis model was constructed by integrating the FBM and UTAUT model frameworks with the aim of exploring the key factors influencing female university students’ willingness and behavior to use health management apps. In this model, attitude and perceived risk are regarded as motivation and ability in FBM, while the four core variables of the UTAUT model (performance expectancy (PE), effort expectancy (EE), social influence (SI) and facilitating conditions (FC)) are introduced. Based on this model, several hypotheses are proposed.

### 2.2 Research hypothesis

The role of social influence (SI) in technology acceptance and use has been a topic of considerable interest in the literature (Venkatesh et al., 2003). Social influence can be defined as the extent to which individuals consider the opinions of others, such as family, friends, or colleagues, to be a factor in their decision-making process. In the context of mHealth, positive feedback from one’s social circle has been shown to positively influence an individual’s willingness to adopt a particular technology (Gumasing et al., 2024; Nie et al., 2023). Research has shown that support from groups in the surrounding environment positively influences individuals‘behavioral choices, and the more positive influences, the stronger the users’ intention to use (Zhu et al., 2023). Furthermore, social influence not only affects individual’s behavioral intention, but also has a significant impact on individual’s perceived risk. The study revealed that when users perceived tangible and evaluated support from mobile social media sites, their uncertainty about the health information they sought from the sites decreased (Deng and Liu, 2017). In other words, the provision of support from the surrounding group can, to some extent, enhance an individual’s perception of the benefits associated with a given situation and reduce the perception of risk associated with the disclosure of private information (Gong et al., 2019). The social cognitive theory (Bandura, 1986) posits that an individual’s attitudes and behaviors are significantly influenced by the people and environments surrounding them. Li et al. found that when an individual’s social circle emphasizes the importance of health management, the individual is more likely to develop positive health attitudes (Li and Wu, 2020). Based on these theoretical supports, it can be posited that there is a positive correlation between social influence and both the willingness to utilize health management applications and the perception of risk with regard to health attitudes.

Therefore, the following assumptions are made in this study:

*H1a*: Social influence will have an important impact on the behavioral intention of female college students to use health management apps.

*H1b*: Social influence will have an important impact on female college students’ perceived risk when using health management apps.

*H1c*: Social influence will have an important impact on female college students’ attitudes of using health management apps.

Performance expectancy (PE) is defined as the extent to which an individual anticipates that utilizing a technology will enhance their work or life performance (Venkatesh et al., 2003). It is regarded as a pivotal determinant influencing user acceptance of health technologies (Xu et al., 2024). Performance expectancy in this study refers to the extent to which individuals feel that using a health management app will help them improve their health. Some studies have found that when users perceive that m-Health apps are effective in helping them manage their health or improve their quality of life, their attitudes towards the technology are usually more positive (Palos-Sanchez et al., 2021), and their willingness to use it typically increases as well (Yang et al., 2024). In addition to the performance and reliability of the technology itself, its effectiveness is also closely linked to the degree of risk perceived by users, whose acceptance of the technology tends to influence their assessment and perception of potential risks. Individuals who believe they have the capacity to control outcomes or are willing to assume risks for potential gains tend to exhibit more favorable attitudes toward risk (Byrne et al., 2016).

Therefore, the following assumptions are made in this study:

*H2a*: Performance expectancy will have an important impact on the behavioral intention of female college students to use health management apps.

*H2b*: Performance expectancy will have an important impact on female college students’ perceived risk of using health management apps.

*H2c*: Performance expectancy will have an important impact on female college students’ attitudes when using health management apps.

Effort expectancy (EE) can be defined as the level of effort that an individual believes is required to utilize a specific technology (Venkatesh et al., 2003). In this study, it refers to the ease with which a user can employ a health management application. This concept is closely related to the user’s perceived ease of use, reflecting the user’s expectation of how straightforward the technology is to learn, use, and interact with. The UTAUT model highlights that effort expectancy is one of the key variables influencing the intention to use a technology (Dwivedi et al., 2016). Effort expectancy has been identified as a significant factor influencing the intention to use a technology, particularly during the initial stages of adoption (Venkatesh et al., 2003). Ooi et al. also indicated that user acceptance of mHealth is closely associated with their perceived effort expectancy (Ooi et al., 2024). Furthermore, Taylor and Todd posited that when users perceive a system as easy to use, they tend to hold a more positive attitude towards it (Taylor and Todd, 1995). Similarly, Featherman et al. argued that users’ perceptions of the ease of use of e-services may influence their perceptions of the risks associated with their use (Featherman and Pavlou, 2003). They proposed that when users perceive that an e-service is easy to operate and understand, they tend to feel more confident, which can significantly reduce their concerns about potential risks. Conversely, if users encounter difficulties in interacting with the service or perceive its navigation and functionality as lacking sufficient intuitiveness, they may become skeptical about the security and reliability of the service, thus increasing their perceived risk of using it.

Therefore, the following assumptions are made in this study:

*H3a*: Effort expectancy will have a significant impact on female college students’ behavioral intention to use health management apps.

*H3b*: Effort expectancy will have a significant impact on the attitudes of female college students when using health management apps.

*H3c*: Effort expectancy will have a significant effect on the perceived risk of female college students in using health management apps.

Facilitating conditions (FC), defined as the degree to which an individual perceives that the organizational and technological infrastructure supports the use of the system, represent a significant predictor of new technology adoption (Venkatesh et al., 2003). Teo and Zhou conducted a study to investigate the impact of facilitating conditions on the attitudes of college students towards technology (Teo and Zhou, 2014). They posited that when users are provided with adequate support, they exhibit positive attitudes towards the technology in question and believe that it will be easier to use. Additionally, Gu et al. emphasized the critical importance of users having access to appropriate conveniences, such as the provision of hardware, software, technical support, online tutorials, and demonstrations, in fostering the intention to use new technology (Gu et al., 2021). Venkatesh et al. stated that conveniences not only enhance users’ attitudes towards technology, but also directly influence their intention to use it, particularly in the context of new information systems (Venkatesh et al., 2012). It is evident that a well-developed technical support system significantly increase users’ intention to use. Furthermore, Collier and Sherrell (2010) indicated that when users perceive the convenience of locating and executing self-help transactions, the simplicity of initiating a transaction, the reduction of uncertainty in the process, and the mitigation of potential risks—whether social or financial—it contributes to a more favorable attitude towards the technology (Collier and Sherrell, 2010).

Therefore, the following assumptions are made in this study:

*H4a*: Facilitating conditions will have an important impact on female college students’ attitudes when using health management apps.

*H4b*: Facilitating conditions will have an important impact on the behavioral intention of female college students to use health management apps.

*H4c*: Facilitating conditions will have an important impact on female college students’ perceived risk of using health management apps.

Perceived risk (PR) refers to the level of risk that an individual perceives when using a health management app (Cocosila and Archer, 2010). It is the cognitive and subjective feeling, influenced by various factors, including psychological, social, and cultural elements (Slovic, 1987). Nowadays there is a great concern about the privacy and security of personal data. Cocosila, et al. found that perceived psychological risk, perceived financial risk, and perceived privacy risk were associated with the overall risk of using mobile information and communication technologies for health promotion interventions (Cocosila and Archer, 2010). Ariff et al. investigated the influence of four types of risk on consumer attitudes towards online services and found that product risk, financial risk, and undeliverability had a negative impact on consumer attitudes (Ariff et al., 2014). Furthermore, perceived risk has been shown to significantly influence the willingness to use management applications (Zhang et al., 2019). The awareness of potential risks, such as personal data leakage, can reduce trust in mHealth services, which in turn hinders the likelihood of their adoption (Deng et al., 2018).

Therefore, the following assumptions are made in this study:

*H5a*: Perceived risk will have an important impact on female college students’ attitudes when using health management apps.

*H5b*: Perceived risk will have an important impact on the behavioral intention of female college students to use health management apps.

Attitude toward behavior (AT) refers to the degree to which a person evaluates or judges the behavior in question (Ajzen, 1991), which, in this study, refers to the user’s evaluation of the health management app. Behavioral intention (BI) is an individual’s tendency or intention to perform a certain behavior, reflecting their desire and willingness to act (Venkatesh et al., 2003). In this study, it refers to the willingness of female college students to use the health management app. According to existing literature, behavioral intention, which is relatively easy to measure and influenced by perception, often translates into actual behavior (AB) (Ajzen, 1991; Yang et al., 2024). In media strategies for promoting health behavior, an individual’s perception of their health can influence their motivation to engage in health-promoting behaviors (Redland and Stuifbergen, 1993).

Therefore, the following assumptions are made in this study:

*H6*: Attitude will have an important impact on female college students’ behavioral intention to use health management apps.

*H7*: Behavioral intention has a significant impact on the actual behavior of female college students using health management apps.

**Figure 1:**
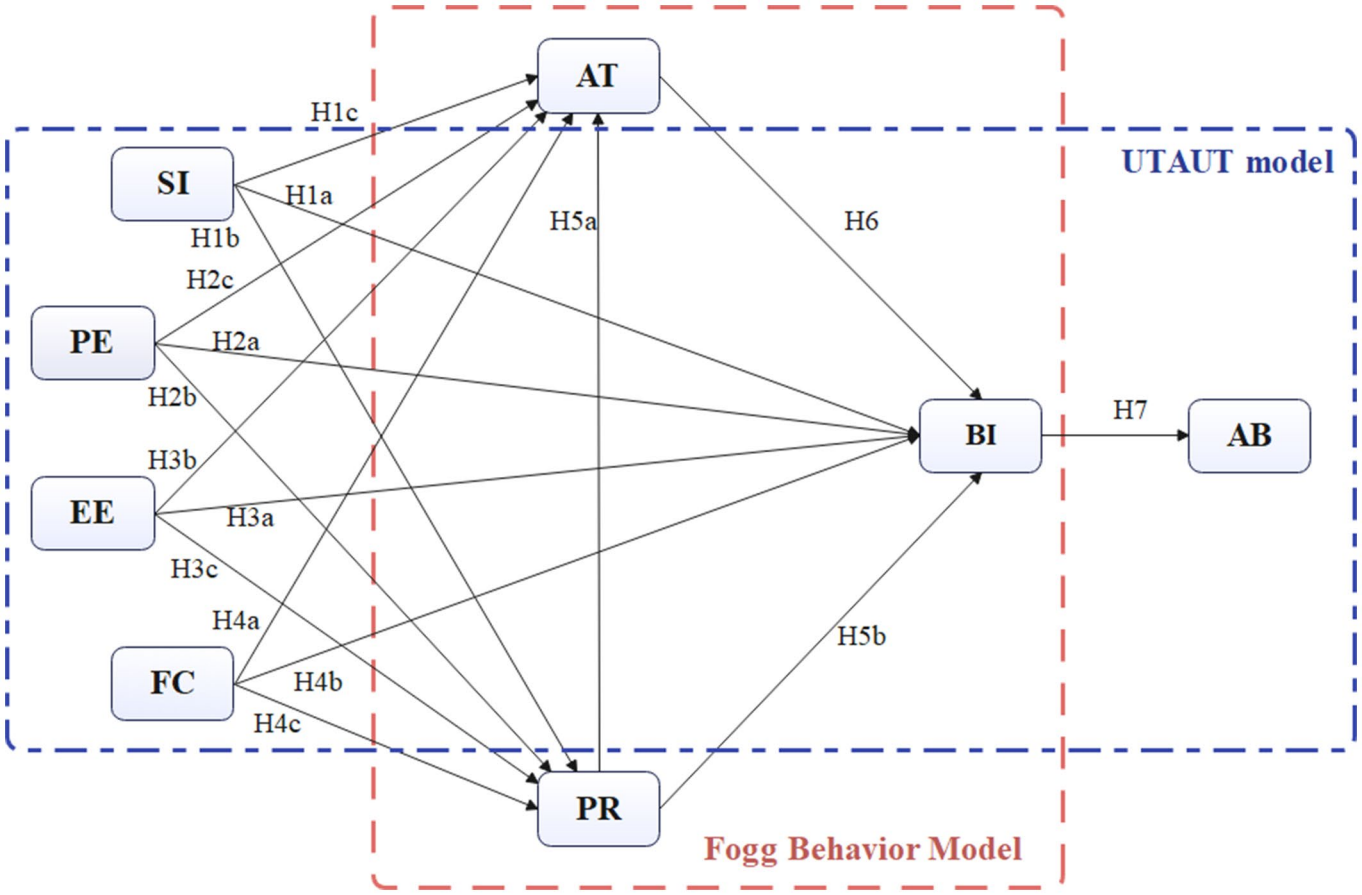
Hypothetical conceptual framework.

Based on the above theories and related studies, this research constructs a theoretical model as shown in Figure 1.

### 2.3 Scale design

The questionnaire is divided into two parts. The first part collects demographic information, including basic characteristics such as gender, major, and year. The second part consists of eight core constructs: social influence, performance expectancy, effort expectancy, facilitating conditions, attitude, perceived risk, behavioral intention, and actual behavior. Each construct contains six to nine questions designed to measure female students’ willingness to use health management applications and the factors influencing it.

In the questionnaire design process, we referred to the definitions of relevant variables in classic literature and to validated questionnaire instruments from existing studies (Ajzen, 1991; Collier and Sherrell, 2010; Venkatesh et al., 2003). However, the pre-survey revealed that simply reproducing the existing questionnaires could not fully adapt to the context and needs of this study. Therefore, the research team made several revisions to the questionnaire to optimize the formulation and structure of the questions, ensuring that the questionnaire accurately reflected the study’s objectives and achieved a high degree of validity and reliability.

To assess the internal consistency of the scales, we calculated the Cronbach’s alpha coefficients, where a value greater than 0.7 is generally considered to indicate acceptable reliability. Additionally, we evaluated the structural reliability of the scales using composite reliability (CR). Following the recommendations of Hair et al. (2010) and Fornell and Larcker (1981), CR values should exceed 0.6. CR values were calculated by dividing the sum of the squared standardized factor loadings by the total variance (including error variance), using the following formula:


$$CR=\frac{{\left(\sum \lambda_{i}\right)}^{2}}{{\left(\sum \lambda_{i}\right)}^{2}+\sum \theta_{i}}$$

λ _i_ = standardized load per project, θ _i_ = error variance for each item.

Meanwhile, convergent validity is used to assess the convergence or correlation of multiple measures within the same dimension. According to the relevant literature, factor loadings should be greater than 0.7, composite reliability should exceed 0.6, and the average variance extracted (AVE) should be greater than 0.5 to ensure that the scale has good convergent validity.

During the pre-survey phase, we analyzed items with low Corrected Item-Total Correlation (CIT) values. If the CIT value of an item was less than 0.5 and the Cronbach’s alpha coefficient increased after the deletion of the item, we removed it to improve the internal consistency of the scale. To ensure measurement validity for each dimension, some item statements were redesigned, and at least three measures were retained for each dimension. All redesigned items were based on a five-point Likert scale, ranging from “strongly disagree” to “strongly agree,” scored from 1 to 5. Measures for each dimension were calculated by averaging the scores of the items within that dimension. After repeated adjustments and validation, the final questionnaire demonstrated good internal consistency, structural reliability, and convergent validity.

### 2.4 Inclusion and exclusion criteria

In order to ensure the validity of the data and minimize errors, the following data inclusion and exclusion criteria are set:

1. The questionnaire was open to female students. Through gender-related questions, we screened for female participants who met the study criteria and excluded data from male participants to ensure that the survey focused on women’s willingness to use health management apps.
2. This study aimed to explore the factors influencing female students’ willingness to use health management apps. Therefore, the responses of faculty and staff were excluded from the analysis. A question regarding professional status was included in the questionnaire to exclude faculty and staff data, retaining only valid responses from current students.
3. The study focused on female students who had experience using health management apps. A screening question was included to determine whether participants had used such apps, and data from those who had not were excluded to ensure relevance.
4. Data from participants with missing information, incomplete responses, or failure to provide key details were excluded. Setting criteria for questionnaire completion and data completeness ensured that the data retained was of analytical value and avoided compromising the study’s results due to missing information.

### 2.5 Data collection

Before the formal survey, a pre-survey of 150 female students using health management apps was conducted via an online platform to assess the applicability of the questionnaire and optimize the survey items. Based on the feedback from the pre-survey, some questions were revised to ensure that they effectively reflected the research objectives and improved the quality and reliability of the questionnaire.

The formal survey was stratified and randomized to ensure that it was not limited to tech-savvy female undergraduates. A total of 800 female undergraduates from various colleges and universities were invited to participate through both online and offline channels. To ensure a diverse sample, the study population included female students ranging from freshman year to graduate school. An initial offline distribution of 325 paper questionnaires was conducted to assess data quality, achieving a response rate of over 85%. After this data quality assessment, the remaining questionnaires were distributed.

**Figure 2:**
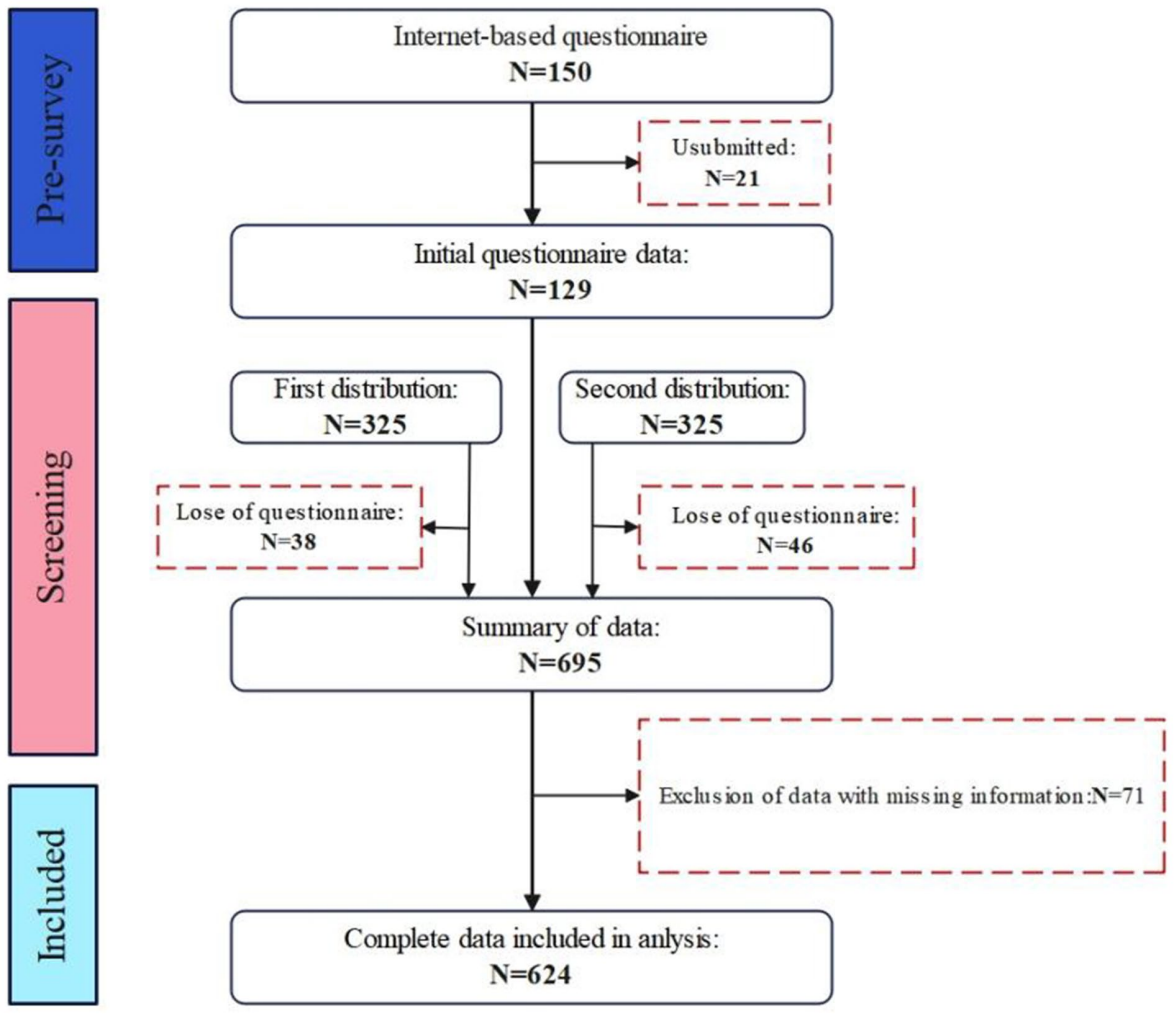
Experimental design and sample selection.

In this study, a total of 695 questionnaires were returned during the data collection phase. Invalid questionnaires were first excluded based on the inclusion/exclusion criteria, such as those from male respondents, incomplete questionnaires, or those with unreasonably short response times. Second, missing values were screened; for a small number of missing values (<5%), the mean interpolation method was used, while questionnaires with a large number of missing values were directly excluded. Ultimately, 624 valid questionnaires were included, resulting in a validity rate of 78%, which met the “10-fold rule” for the minimum sample size requirement in structural equation modeling (SEM). Informed consent for this study was obtained from all participants and/or their legal guardians (Figure 2).

### 2.6 Statistical analysis tools

This study employed a variety of statistical tools to ensure the rigor and scientific validity of the data analysis. First, Excel was used for data management, and missing data (<5%) was handled using mean interpolation, while larger amounts of missing data were excluded. Next, demographic characterization and correlation analysis were performed using SPSS 23.0 to describe the sample characteristics and explore the relationships between variables. Based on the FBM and UTAUT models, structural equation modeling (SEM) was conducted using AMOS 24.0 to quantify the causal relationships between latent variables. During the model fit assessment, the fit of the model to the data was verified by calculating fit indices (e.g., *X*^2^/df, RMSEA, etc.). Factor loadings (i.e., standardized regression coefficients) between latent constructs and observed variables were then calculated using AMOS 24.0, and path coefficients, composite reliability (CR), and significance *p*-values (*p* < 0.05) were reported. Finally, the key factors influencing college women’s willingness to use health management applications were identified.

## 3 Result

### 3.1 Demographic characterization

As shown in Table 1, among the female college students in a medical university investigated in this study, most of them are third-year students (21.1%), followed by postgraduate students and above (19.9%). The largest number of people majored in medicine (14.6%). Most people have an average income of 1,500–1999 (36.9%), and only 13% have an average income of more than 2,500. It can be seen that the expenditure of female college students will be subject to certain economic restrictions. Most people spend 100–149 on health (21.2%). Hundred and thirty seven people have moderate learning intensity (57.7%). The weekly activity of most students is less than 2 h (42.6%). The most commonly used health management app is KEEP (36.2%). And 82.7% of female college students choose to use it on a weekly basis. The majority had a BMI of 18.5–23.9 (63.5%), indicating that most of the surveyed population was in the normal range.

**Table 1:** Demographic characterization.

Category	Option	Frequency	Percent	Category	Option	Frequency	Percent
Grade	Freshman	89	14.3	Weekly activity	<2 h	266	42.6
	Sophomore	113	18.1		2–5 h	161	25.8
	Third-year students	132	21.1		6–9 h	106	17.0
	Fourth-year students	99	15.9		10–13 h	57	9.1
	Fifth-year students	67	10.7		14–17 h	15	2.4
	Postgraduate and above	124	19.9		≥18 h	19	3.0
Major	Science	78	12.5	Weekly duration of use	Yes	516	82.7
	Engineering	61	9.8		No	108	17.3
	Medicine	91	14.6	Commonly used health management app	Ping’an Healthcare	47	7.5
	Management	72	11.5		Dr. Lilac	146	23.4
	Arts	37	5.9		Mint Health	54	8.7
	Economics	86	13.8		Meiyu	104	16.7
	Law	59	9.5		KEEP	226	36.2
	Education	47	7.5		Others	47	7.5
	Literature	52	8.3	BMI	<18.5	98	15.7
	Others	41	6.6		18.5–23.9	396	63.5
Income	Below 500	6	1.0		24–27.9	76	12.2
	500–999	25	4.0		≥28	54	8.7
	1,000–1,499	175	28.0				
	1,500–1999	230	36.9				
	2000–2,499	107	17.1				
	2,500–2,999	26	4.2				
	3,000 and above	55	8.8				
Health expenditure	Below 10	94	15.1				
	10–49	89	14.3				
	50–99	115	18.4				
	100–149	132	21.2				
	150–199	58	9.3				
	200–249	46	7.4				
	250–299	29	4.6				
	300 and above	61	9.8				
Learning Intensity	low	144	23.1				
	Medium	360	57.7				
	High	120	19.2				

### 3.2 Reliability and validity tests

The test results of this study are shown in Table 2. Cronbach’s Alpha and CR values in all aspects of this study are higher than the recommended values, indicating that the model had good internal consistency.

**Table 2:** Validity of model convergence.

Facet	Item	Parameter significance estimation				Factor loading	Cronbach’s α	Composition validity	Convergence validity
		Unstd	S.E.	C.R.	P	Std		CR	AVE
SI	v1	1				0.718		0.8909	0.5387
	v2	1.095	0.052	21.046	***	0.76			
	v3	0.975	0.048	20.341	***	0.741			
	v4	1				0.705	0.891		
	v5	1.061	0.051	20.72	***	0.751			
	v6	1.115	0.055	20.438	***	0.744			
	v7	0.934	0.048	19.486	***	0.717			
PE	v8	1				0.753		0.9062	0.5801
	v9	0.959	0.05	19.04	***	0.756			
	v10	0.892	0.047	19.106	***	0.758			
	v11	0.955	0.052	18.373	***	0.732	0.906		
	v12	0.935	0.046	20.292	***	0.8			
	v13	0.888	0.046	19.193	***	0.761			
	v14	0.92	0.047	19.434	***	0.77			
EE	v15	1				0.803		0.9216	0.6273
	v16	0.936	0.049	19.212	***	0.71			
	v17	0.906	0.042	21.432	***	0.772			
	v18	1.03	0.045	23.145	***	0.818	0.921		
	v19	1.046	0.043	24.314	***	0.847			
	v20	0.978	0.045	21.67	***	0.779			
	v21	0.969	0.042	22.794	***	0.808			
FC	v22	1				0.775		0.9054	0.5778
	v23	0.978	0.046	21.314	***	0.805			
	v24	0.937	0.047	19.94	***	0.761			
	v25	0.9	0.047	18.983	***	0.73	0.905		
	v26	0.921	0.049	18.913	***	0.728			
	v27	0.97	0.048	20.046	***	0.765			
	v28	0.948	0.048	19.715	***	0.754			
AT	v29	1				0.591		0.8448	0.3778
	v30	1.016	0.082	12.414	***	0.61			
	v31	1.091	0.084	12.994	***	0.649			
	v32	1.12	0.083	13.42	***	0.68			
	v33	0.91	0.077	11.841	***	0.573	0.932		
	v34	1.013	0.085	11.849	***	0.573			
	v35	1.059	0.081	13.044	***	0.653			
	v36	1.013	0.085	11.976	***	0.581			
	v37	1.06	0.085	12.441	***	0.612			
PR	v38	1				0.682		0.9062	0.5819
	v39	0.965	0.06	15.965	***	0.696			
	v40	0.984	0.062	15.767	***	0.687	0.909		
	v41	1.227	0.065	18.973	***	0.845			
	v42	1.239	0.067	18.414	***	0.816			
	v43	1.243	0.065	19.249	***	0.859			
	v44	1.002	0.06	16.698	***	0.731			
BI	v45	0.864	0.053	16.436	***	0.665	0.924	0.8634	0.5137
	v46	0.895	0.053	17.025	***	0.687			
	v47	0.897	0.052	17.244	***	0.695			
	v48	0.925	0.049	18.781	***	0.752			
	v49	0.945	0.051	18.392	***	0.738			
	v50	1				0.758			
AB	v51	1				0.749	0.914	0.8736	0.5333
	v52	0.979	0.056	17.377	***	0.709			
	v53	1.157	0.064	18.163	***	0.74			
	v54	1.127	0.064	17.544	***	0.716			

***p < 0.001.

Validity is a measure of whether a comprehensive evaluation system can accurately reflect the purpose and requirements of the evaluation. And it means that the accuracy of the characteristics can be measured by means of measuring tools. The higher the validity, the better the measurement results can indicate the characteristics to be measured, and vice versa, the lower the validity. The results show that discriminant validity (DV) can be used to quantify concepts that are not conceptually related to each other. Discriminant validity aims to show any evidence of discrimination based on differences between all components. As can be seen from the figure, the AVE values on the diagonal are all greater than 0.5 and greater than the values below the diagonal. This proves that the validity of the model in this study is relatively high. The test results of this study are shown in Table 3.

**Table 3:** Convergent validity test results.

Variables	AVE	ASI	APE	AEE	AFC	AAT	APR	ABI	AAB
ASI	0.5387	1							
APE	0.5801	0.812**	1						
AEE	0.6273	0.769**	0.819**	1					
AFC	0.5778	0.788**	0.759**	0.801**	1				
AAT	0.3778	0.838**	0.813**	0.827**	0.854**	1			
APR	0.5819	0.388**	0.350**	0.361**	0.325**	0.352**	1		
ABI	0.5137	0.656**	0.679**	0.703**	0.709**	0.729**	0.259**	1	
AAB	0.5333	0.752**	0.768**	0.760**	0.741**	0.783**	0.307**	0.770**	1

***p < 0.001, **p < 0.01, *p < 0.05.

### 3.3 Model measurements

#### 3.3.1 Model fit goodness-of-fit test

**Figure 3:**
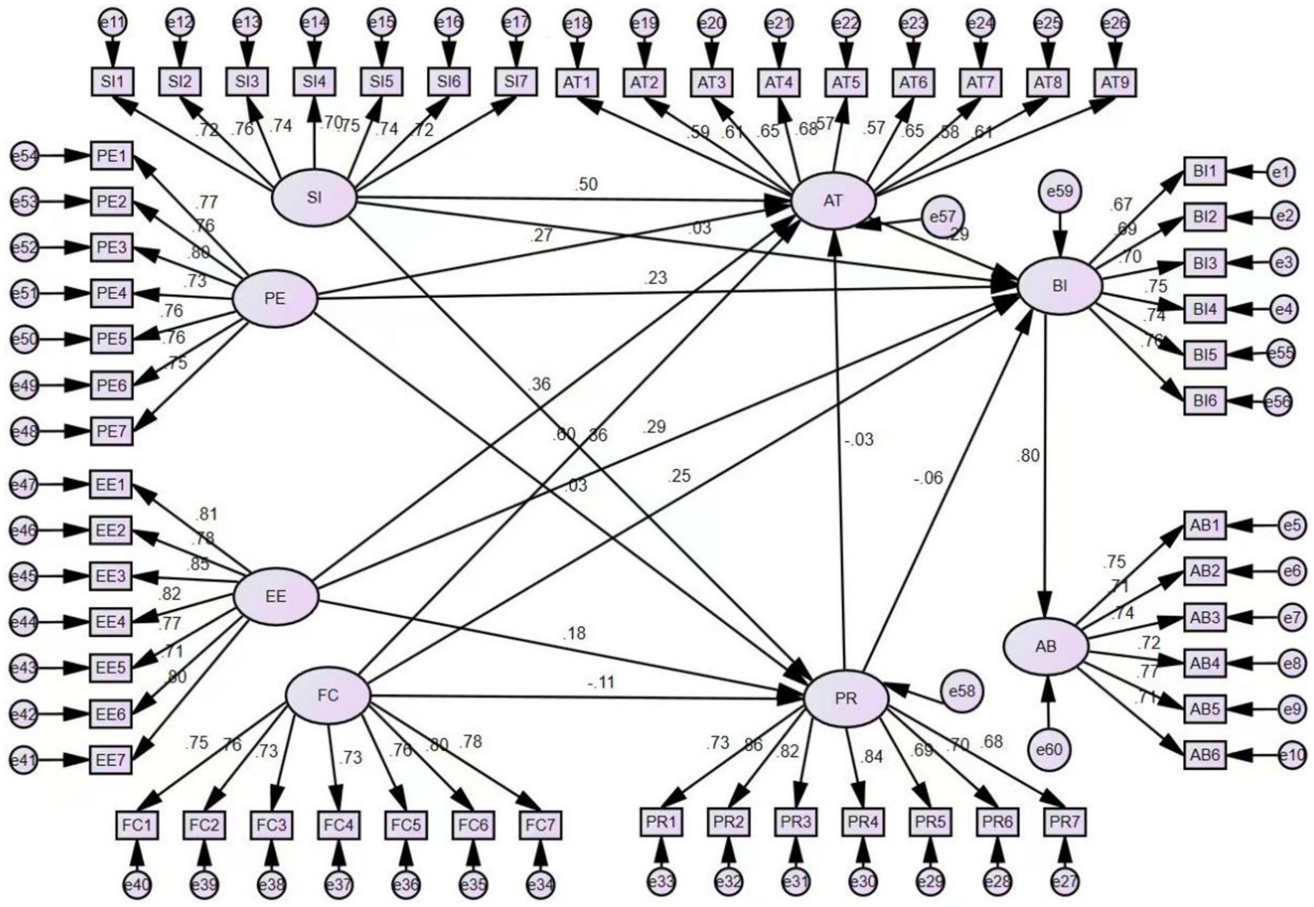
UTAUT-FOGG model (indicators of model fit: X^2^/DF = 4.712, RMSEA = 0.077, PGFI = 0.648, PNFI = 0.731, PCFI = 0.768).

The UTAUT model based on Fogg theory has a good ability to measure the willingness of female college students to use health apps, in terms of the absolute fitting metrics, the value of *X*^2^/df is 4.712, which is less than 5. The value of RMSEA is 0.077, which is less than 0.08. The absolute fit metrics are ideally suited. In terms of the parsimonious fitting metrics, the value of PGFI is 0.648, which is greater than 0.5. The value of PNFI is 0.731, which is greater than 0.5. The value of PCFI is 0.768, which is greater than 0.5. The parsimony fit indicators are well adapted. Overall, the indicators of the scale fit well and the model fit well, as shown in Figure 3.

#### 3.3.2 Model results

As shown in Table 4 and Figure 3, Social Influence (SI), Performance Expectancy (PE), Effort Expectancy (EE), and Facilitating Conditions (FC) have positive relationships with User Attitudes (AT), so hypotheses H1, H4, H7, and H10 are valid (*β* = 0.497, *p* < 0.001; *β* = 0.268, *p* < 0.001; *β* = 0.359, *p* < 0.001; *β* = 0.603, *p* < 0.001). Social Influence (SI) and Effort Expectancy (EE) have positive relationships with Perceived Risk (PR), Facilitating Conditions (FC) have negative relationship with Perceived Risk (PR), supporting hypotheses H3, H9, and H12 (*β* = 0.36, *p* < 0.001; *β* = 0.183, *p* < 0.001; *β* = −0.108, *p* < 0.01). And Health Expectancy (PE), Effort Expectancy (EE), Facilitating Conditions (FC) and Attitude (AT) have a positive relationship with Behavioral Intention (BI), supporting hypotheses H5, H8, H11, and H14 (*β* = 0.231, *p* < 0.001; *β* = 0.285, *p* < 0.001; *β* = 0.25, *p* < 0.01; *β* = 0.291, *p* < 0.05). Behavioral intention (BI) had a positive correlation with Actual Behavior (AB), supporting hypothesis H16 (*β* = 0.804, *p* < 0.001).

**Table 4:** Structural model assessment results for direct relationships.

Path	Non-standardized path	Standardized path	S.E.	C.R.	P	Conclusion
AT <--- SI	0.311	0.497	0.027	11.327	***	Support
AT <--- PE	0.148	0.268	0.018	8.004	***	Support
AT <--- EE	0.212	0.359	0.022	9.806	***	Support
AT <--- FC	0.357	0.603	0.029	12.508	***	Support
AT <--- PR	−0.016	−0.027	0.019	−0.845	0.398	Not-Support
PR < --- SI	0.372	0.36	0.046	8.011	***	Support
PR < --- PE	0.031	0.034	0.037	0.84	0.401	Not-Support
PR < --- EE	0.179	0.183	0.04	4.433	***	Support
PR < --- FC	−0.106	−0.108	0.04	−2.642	**(0.008)	Support
BI <--- SI	0.032	0.035	0.065	0.486	0.627	Not-Support
BI <--- PE	0.186	0.231	0.039	4.726	***	Support
BI <--- EE	0.246	0.285	0.049	4.998	***	Support
BI <--- FC	0.216	0.25	0.07	3.08	**(0.002)	Support
BI <--- PR	−0.051	−0.058	0.035	−1.455	0.146	Not-Support
BI <--- AT	0.424	0.291	0.17	2.502	*(0.012)	Support
AB <--- BI	0.769	0.804	0.049	15.737	***	Support

***p < 0.001, **p < 0.01, *p < 0.05.

But the effects of Social Influence (SI) and Perceived Risk (PR) on Behavioral Intention (BI) (*β* = 0.035, *p* = 0.627; *β* = −0.058, *p* = 0.146) are not statistically significant. The effect of Perceived Risk (PR) on Attitude (AT), Health Expectancy (PE) on Perceived Risk (PR) (*β* = −0.027, *p* = 0.398; *β* = 0.034, *p* = 0.401) are not statistically significant either.

## 4 Discussion

Focusing on a population of female college students, this study skillfully blends the FBM with the UTAUT Model to construct a comprehensive and effective theoretical framework that explores various psychological and social factors influencing the use of health management applications, thereby offering valuable insights into understanding and improving the adoption of these systems.

### 4.1 Factors influencing attitude

Based on the results of structural equation modeling, this study shows that social influence, performance expectancy, effort expectancy, and facilitating conditions all have a significant effect on attitude, and hypotheses H1c, H2c, H3b, and H4a are supported. However, perceived risk has no significant effect on attitude.

Of these, facilitating conditions had the most significant effect on attitude. This is consistent with Wu et al.’s finding that good facilitating conditions are factors that influence older adults to develop positive attitudes toward health and exercise (Wu et al., 2016). Based on the content of the convenience dimension, good facilitating conditions (such as device compatibility, ease of use, and abundant help resources) make it easier for individuals to access and use such applications, thus contributing to their positive attitudes. This finding further suggests that the provision of good resources and technical support is a key factor in improving user attitudes, especially in technological application scenarios, where improving facilitating conditions can help reduce user resistance.

Second, the impact of social influence on attitude is also more significant. By referencing the relevant items related to social influence in the questionnaire, such as SI1 and SI3, it was found that female college students’ attitudes toward selecting health management apps are more likely to be influenced by their peers and online communities. Social influence may lead them to adopt a more positive attitude toward this technology. This is similar to the findings of Dunn et al. (2001). Therefore, it is particularly important to strengthen the social recommendation mechanisms within the application. By incentivizing users to share their insights and experiences with health management applications, not only does it increase interaction among users, but it also helps build a positive community around the app. Additionally, partnering with experts or celebrities in the health field and leveraging their influence to promote the app on social media can effectively enhance its credibility and appeal. This, in turn, improves users’ positive attitudes toward the health management app, ultimately encouraging actual usage behavior. Such a strategy not only helps to increase user engagement and satisfaction but also provides a competitive advantage for the app in the marketplace.

The effects of performance expectancy and effort expectancy on attitudes were relatively small, but still significant. According to the data from the performance expectancy and effort expectancy dimensions in the questionnaire, PE3 indicates that most users believe these apps can effectively meet their health needs. Similarly, EE2 shows that users generally find these apps easy to operate, with operational difficulty not being a barrier. This suggests that when users believe that a technology is not only effective in meeting their needs (performance expectancy), but also does not cause too much hassle to use (effort expectancy), they are significantly more likely to develop a positive attitude toward the technology (Wang et al., 2023). This positive attitude is an important catalyst that drives users to take action. It not only promotes the initial adoption of the technology but also increases user satisfaction and loyalty throughout the usage process. For female college students, if they expect to improve their health by using a health management app and perceive that the effort required to use the app is low, this expectation may lead them to develop a more positive attitude toward their health (Palos-Sanchez et al., 2021). Therefore, app developers need to enhance health feedback mechanisms to provide users with clear tracking and feedback on their health improvement progress, ensuring a positive experience during use. At the same time, well-designed user interfaces, intuitive operations, and effective performance can enable technologies to better meet users’ expectations, thus creating a positive image in users’ minds. Such a technology not only attracts users but also generates positive word-of-mouth within the user community, further expanding its reach and use.

In addition, this study found no significant relationship between perceived risk and users’ attitudes toward health management apps. However, many studies have shown that perceived risk usually has a negative impact on users’ attitudes toward technology adoption (Sinha et al., 2021; Xie et al., 2017). This may be due to the fact that, in the context of this study, users are likely not concerned about the security and reliability of data or services based on the perceived risk dimension. The findings suggest that users’ overall perception of risk is relatively low. Many applications are designed with enhanced privacy and security safeguards, which reduce users’ risk perceptions through transparent user agreements, data encryption, etc. (Robles et al., 2020). Moreover, the student population has become accustomed to relying on digital technologies in all aspects of their lives (Yot-Domínguez and Marcelo, 2017), and this familiarity and dependence have led to a higher level of tolerance and trust in the security of digital applications, thus reducing the impact of privacy and security concerns on their attitudes toward use.

### 4.2 Factors influencing behavioral intention

This study shows that attitude, effort expectancy, facilitating conditions, and performance expectancy all have a significant effect on the intention to use health management applications, and hypotheses H7, H3a, H4a, and H2a are valid. However, social influence and perceived risk have no significant direct effect on behavioral intention.

Specifically, attitude has the most significant effect on the intention to use, which is consistent with the Technology Acceptance Model (TAM), suggesting that a user’s positive attitude toward a technology directly increases their intention to use it (Davis, 1989). Based on the content of the attitude dimension in the questionnaire, most users agree with the potential health benefits of health management apps. Therefore, when users have positive attitudes toward health management applications, they are more likely to adopt and continue using the technology. Identification with and satisfaction with these apps are also key factors that drive users’ willingness to use them (Bellary et al., 2024), suggesting that fostering positive attitudes toward health management apps is critical to increasing usage.

Second, effort expectancy also had a significant effect on intention to use, suggesting that effort expectancy plays a key role in shaping users’ intention to use health management APPs (Ooi et al., 2024). When users perceive that using the category app is both intuitive and convenient, and does not require too much time and effort, they are more likely to develop a positive intention to use it (Cao et al., 2022; Sahoo et al., 2023). This perceived ease of use not only reduces users’ psychological barriers, but also increases their perception of the value of the application, which motivates them to try and continue using it. In this study, effort expectancy is reflected through multiple items. For example, EE2 indicates that most users find using these apps straightforward, while EE4 shows that users generally perceive the interface of health management apps as easy to use. In short, a user-friendly interface and a smooth user experience are important factors that promote the widespread adoption of health management apps (Tao and Liu, 2024).

Facilitating conditions have a positive effect on behavioral intention. According to the content of this study, the importance of facilitating conditions reflects users’ dependence on external support when using health management applications, especially in terms of technical support and platform compatibility, which increase users’ intention to use. Yang et al. also pointed out that facilitating conditions are an important factor that influences the intention and actual behavior of mHealth users in Indonesia (Yang et al., 2024). Therefore, reducing technical barriers and improving the usability of technology and devices, along with providing effective customer support and tutorials, will not only enhance the user experience but also promote greater user participation and long-term use.

Performance expectancy has a positive effect on behavioral intentions. This means that users are more inclined to use health management apps when they expect to gain health improvements or other useful outcomes through the use of the technology, and Yuan, S. et al. reported similar results in a study on fitness apps (Yuan et al., 2015). When individuals believe that health management apps are effective in promoting or maintaining their health status, this belief tends to translate into more frequent use. In this study, PE3 indicates that most users have high expectations regarding the health benefits of the app, while PE6 reflects users’ positive evaluations of the app’s functionality. This positive expectation not only increases their trust in the app, but also significantly increases their willingness to use the app. In other words, users’ perceptions of the potential benefits of the app are important drivers that motivate them to continue to interact and engage deeply. Therefore, in order to stimulate and maintain high levels of user engagement, app developers must continually reinforce users’ positive perceptions of the health benefits of the app and ensure that the app’s features and services meet users’ health needs and expectations.

The effects of perceived risk and social influence on intention to use are insignificant, but they are worthy of further investigation. First, the insignificant effect of perceived risk on usage intention may be due to users’ increased trust in health management apps, and increased trust can reduce perceived risk (Jiang et al., 2022). With the adoption of protective measures, consumers are more confident in engaging with new technologies, which may mitigate their security concerns to some extent (Gurung and Raja, 2016). In addition, it has been mentioned that many users are less aware of privacy and personal data protection issues when using Internet services, and they often unintentionally disclose personal information without realizing the privacy risks (Soumelidou and Tsohou, 2021). Particularly for the younger generation of users, such as the student population, the perceived risk dimension in the questionnaire reveals that their concerns about issues like privacy breaches and data security may be relatively limited. Therefore, the importance of perceived risk in influencing their willingness to use the app may be diminished.

In addition, this study found that social influence did not significantly affect the willingness to use, and some scholars have reported similar results in their studies. For example, Venkatesh et al. found that listening to others is more important in the early stages of experience, especially in coercive environments, but as experience is gained, normative pressure decreases and an individual’s willingness to use the system is based more on instrumental rather than social factors (Venkatesh et al., 2003). Candra et al. also noted that in certain populations or settings, other factors (e.g., perceived usefulness and usability) may be more influential in determining a user’s behavioral intention to use an mHealth app (Candra et al., 2024). Similarly, Lu et al. found that social influence as a construct did not serve as a determinant of persistent intention, possibly because social influence declines over time (Lu, 2014). Therefore, based on the content of the social influence dimension in this study, it is found that after users develop independent habits of using health management apps, they may rely more on their own experience and judgment, with less influence from the opinions of family and friends, recommendations from medical professionals, or policy guidance.

### 4.3 Factors influencing perceived risk

According to the results of this study, performance expectancy, facilitating conditions, and social influence all have a significant effect on perceived risk, and hypotheses H3c, H4c, and H1b are valid. However, performance expectancy does not have a significant direct impact on perceived risk among college women using health management applications.

Social influence has the greatest impact on perceived risk, suggesting that the opinions of others have the greatest effect on users when assessing the risk of using a health management app (Deng and Liu, 2017). Based on the items in the social influence dimension, the positive impact of social influence on perceived risk found in this study reflects the fact that users who perceive widespread use and support for a technology from external sources (e.g., friends, family, or the social environment) may actually have greater concerns about the safety and privacy of that technology. This phenomenon is particularly notable in the context of health management applications. Due to the sensitive nature of health data, users may be concerned about whether they will experience problems such as privacy violations while using the technology, despite widespread social support for it. Similar findings suggest that social influence may sometimes increase users’ perceptions of risk, especially if they believe that widespread use of the technology could heighten security concerns (Knoll et al., 2015).

Second, the positive relationship between effort expectancy and perceived risk suggests that female students tend to perceive higher risk when they believe that using a health management app is easy and requires less effort. Specifically, EE2 indicates that most users find these apps easy to operate. Correspondingly, PR1 reveals that despite the convenience of using the apps, users still have certain concerns about their privacy and security. As Badr mentioned in a study on IoT devices, the increasing ease of use of products is often accompanied by concerns about the vulnerability of stored personal information to breaches and cyberattacks (Badr et al., 2021). Therefore, users may question whether an easy-to-use application has sufficient security measures to protect their personal information from the risk of leakage and cyberattacks.

This study also found a negative effect of facilitating conditions on perceived risk. This is consistent with Deochand, N. et al., who, in developing a decision tool for risk assessment in functional analysis, mentioned that risk in functional analysis (FA) can be reduced when experienced behavioral analysts, medical supervisors, and trained staff assist in the assessment (Deochand et al., 2020). Based on the content of the facilitating conditions dimension, this implies that users perceive their risk to be reduced when using a health management app with adequate technical support and resources. Kim et al. also found that in the healthcare domain, resource availability and technical support increase users’ trust in using the technology (Kim et al., 2024). Therefore, external help, such as adequate resources and technical support, can help users cope with potential problems, thus reducing their concerns about possible technical failures or privacy issues.

The impact of performance expectancy on perceived risk is not significant. Based on the content of the performance expectancy dimension, this indicates that users’ perception of potential risks is not notably influenced by the app’s ability to improve their health or provide health-related knowledge when using health management apps. This may be due to the fact that the perception of risk when using health management apps is influenced more by ease of use and the social environment rather than the functional performance of the system itself (Featherman and Pavlou, 2003). This suggests that even if users find these apps useful for improving health or raising health awareness, they may still maintain a high perception of risk if they are skeptical about the security or privacy of the system.

### 4.4 Factors influencing actual behavior

According to the analysis of the results of H16, behavioral intention is an important factor in predicting the actual behavior of female college students using health management apps. As explored in the Integrated Technology Acceptance and Use Model, an individual’s behavioral intention plays an important role in actual action (Venkatesh et al., 2003). When individuals develop a positive willingness to use a health management app, they are more likely to translate this intrinsic motivation into actual use behavior. Combined with the behavioral intention dimension items in this study, BI1 indicates that most users have a strong intention to continue using the app, while BI2 further demonstrates their positive attitude toward it. This willingness is the key factor that drives them from mere interest to actually downloading, installing, and starting to use the app. Therefore, in order to increase user stickiness and market share of apps, developers need to focus on the key drivers of user willingness.

## 5 Conclusion

By combining the Fogg Behavioral Model with the UTAUT Model for modeling, this study aims to empirically validate the factors that determine female college students’ willingness to use health management applications. The research model shows that social influence, performance expectancy, effort expectancy, and facilitating conditions positively predict attitude; social influence, performance expectancy, and facilitating conditions positively predict perceived risk; and performance expectancy, effort expectancy, facilitating conditions, and attitude positively predict an individual’s intention to use such applications, which in turn affects actual behavior.

This study innovatively combines the Fogg Behavioral Model and the UTAUT Model to construct an integrated model specifically designed to study the behavior of female college students using health management apps. The model distinguishes between internal and external factors and examines how individual motivation and ability influence users’ intention to use and behavior. By introducing perceived risk and attitude variables, the model further enriches the understanding of user behavior. This study not only verifies the validity of the theoretical model, but also provides practical suggestions for the design of health management applications, such as simplifying the operation process and interface, optimizing application functions, establishing user communities, designing a real-time health feedback system, and strengthening privacy management. Moreover, this study provides guidance for the practical application of women’s health management apps, which can help promote the development of women’s health and contribute to improving the quality of life of women.

However, this study was limited by time, geography, and resources. First, a cross-sectional design was used to measure recent health management app use among female college students, which does not reflect changes over time. In the future, longitudinal, multi-stage, and multiple measures could be used to obtain long-term data. Second, the study population consisted primarily of medical students, who may have better health literacy and health-related knowledge, which could influence the study’s results. In the future, the research scope can be expanded to include more individuals from other specialties and regions, as well as individuals of different ages, cultural backgrounds, and health statuses. Additionally, the research population in this study was limited to female college students in China, and future studies could also focus on the willingness of female college students from more countries to use health management apps. Furthermore, many other factors, such as individual factors like habit, hedonic motivation, and personal innovativeness, as well as external factors such as app usability, design, and the broader social environment, may also interact with users’ behavioral intentions in complex ways. Future research could explore the effects of these factors on health app usage intentions. Finally, future research could further examine the influence of technological experience, or how individual differences, such as privacy sensitivity, act as moderating variables to influence the relationship between psychological factors and perceived risk. By analyzing the mechanisms of these moderating variables in depth, more precise strategic suggestions for the design and promotion of health management apps could be provided.

## Data Availability

The raw data supporting the conclusions of this article will be made available by the authors, without undue reservation.
