# Supplementary material for: Analysis of willingness to use health management APP for female college students: application of UTAUT model based on Fogg theory
Source: Front Psychol. 2024 Nov 7;15:1466566. doi: 10.3389/fpsyg.2024.1466566 (PMC11578752; doi:10.3389/fpsyg.2024.1466566)
Supplement: Supplementary file 1 [file Presentation_1.pdf]

APPENDIX

The adapted questionnaire was used to test the acceptability of health management apps among female university students.

Constructs	Item Numbers	Statement
SI	SI1	I have friends and classmates who are using these apps.
	SI2	I have family members, relatives who use these apps.
	SI3	There are many people on the web who use or recommend these apps.
	SI4	These apps were recommended to me by doctors, nurses, and other medical professionals.
	SI5	I will use these apps for policy-related reasons.
	SI6	Using these apps makes me feel like I'm not outdated.
	SI7	If people around me recommend this type of app, I would use it.
PE	PE1	These apps can enrich my health knowledge.
	PE2	These apps can help me learn about health and disease-related information.
	PE3	I believe using these apps basically meets my health needs.
	PE4	These apps can provide me with timely medical services.
	PE5	These apps can help me monitor my health status.
	PE6	Using these apps makes it more convenient to consult with doctors.
	PE7	Using these apps has improved my efficiency in seeking medical care and saved me time.
EE	EE1	I find it easy to understand the services and knowledge provided by these apps.
	EE2	For me, it is easy to use these apps proficiently.
	EE3	I am able to handle the difficulties I encounter while using these apps well.
	EE4	I find health management apps easy to operate.
	EE5	I am able to use these apps independently to access services and knowledge.
	EE6	I find it very easy to use these apps to consult with doctors.
	EE7	I think it's easy to learn how to operate these apps.
FC	FC1	My phone has enough memory to run these apps.

	FC2	My phone has enough data to use these apps.
	FC3	These apps are easy to use and have a clear interface, which makes me more willing to use them.
	FC4	These apps can be used anywhere, anytime, so I prefer to use them.
	FC5	It would make me use these apps better if I had a professional customer service agent to help me solve the problems I encountered in operation.
	FC6	The fact that I have access to the health information and knowledge I want, when and where I want it, makes me more likely to use the program.
	FC7	The doctor's replies are timely and easy to understand, and he can give a clear treatment plan, which will make me more willing to use it.
AT	AT1	I take my health seriously, and the use of these apps in my daily life is valuable.
	AT2	I believe that adherence to health management can improve my health.
	AT3	I enjoy using these apps to improve my health.
	AT4	I'm interested in health management apps that can improve my health.
	AT5	Using these apps to improve your health in your daily life is desirable.
	AT6	Using these apps will help me manage my health and address some of my illnesses.
	AT7	These apps have made me take my health more seriously.
	AT8	In the event of a health problem, I would choose to use these apps to retrieve the problem.
	AT9	I can change bad habits that affect my health with the help of these apps.
PR	PR1	I am concerned that using these apps could result in the leakage of my personal information.
	PR2	I'm concerned that the services offered by these apps are not trustworthy.
	PR3	I don't think the data recorded by these apps is reliable.
	PR4	I doubt the authenticity of the doctors on these apps.
	PR5	I think counseling using an app is less effective than face-to-face clinics.
	PR6	I'm worried about charging a lot on these apps.
	PR7	I'm worried that the money spent on these apps is not worth it.
BI	BI1	I want to continue using health management apps.
	BI2	I would recommend the health management apps to family and friends.
	BI3	I am willing to try to learn to use a health management app.

	BI4	I would use a health management app if I had the chance.
	BI5	I have a positive attitude towards the use of health management apps.
	BI6	If I had access to a health management app, I would be more than willing to use it.
AB	AB1	I would use a health management app to improve my health.
	AB2	I will try to learn to use health management apps.
	AB3	Using health management apps has become a habit for me.
	AB4	I use health management apps a lot.
	AB5	I have health needs, so I will use health management apps.
	AB6	I would recommend health management apps to people around me.
